# Hereditary diffuse gastric cancer in a Japanese family with *CDH1* mutation three case reports

**DOI:** 10.1007/s12672-023-00623-4

**Published:** 2023-01-31

**Authors:** Futoshi Muranaka, Emiko Kise, Shigeo Tokumaru, Masato Kitazawa, Yusuke Miyagawa, Tomoaki Suga, Takeshi Uehara, Mai Iwaya, Shota Kobayashi, Midori Sato, Daisuke Gomi, Hidetaka Yamada, Haruhiko Sugimura, Tomoki Kosho, Yuji Soejima, Tomonobu Koizumi

**Affiliations:** 1 Department of Surgery, Division of Gastroenterological, Transplantation and Pediatric Surgery, Hepato-Biliary-Pancreatic, Shinshu University School of Medicine, Matsumoto, Japan; 2 Center for Medical Genetics, Shinshu University Hospital, Matsumoto, Japan; 3 Endoscopic Examination Center, Shinshu University Hospital, Matsumoto, Japan; 4 Department of Laboratory Medicine, Shinshu University School of Medicine, Matsumoto, Japan; 5 Department of Pathology, Kurashiki Central Hospital, Kurashiki City, Japan; 6 Department of Hematology and Medical Oncology, Shinshu University School of Medicine, 3-1-1, Asahi, Matsumoto, 390-8621 Japan; 7 The First Department of Pathology, Hamamatsu University School of Medicine, Hamamatsu, Japan; 8 Department of Medical Genetics, Shinshu University School of Medicine, Matsumoto, Japan; 9 Research Center for Supports to Advanced Science, Shinshu University, Matsumoto, Japan; 10 Division of Clinical Sequencing, Shinshu University School of Medicine, Matsumoto, Japan

**Keywords:** Hereditary Diffuse Gastric Cancer, E-cadherin, CDH1 mutations, Signet ring cell carcinoma, Esophagogastroduodenoscopy, Random and target biopsies, Total prophylactic gastrectomy

## Abstract

**Background:**

Germline pathogenic variants in the E-cadherin gene *CDH1* cause hereditary diffuse gastric cancer (HDGC), which is an autosomal dominant cancer syndrome, accounting for 1–3% of all gastric cancers. HDGC harboring a *CDH 1* variant is extremely rare in Japan.

**Method:**

In this study we report the clinical courses of three cases with HDGC from a single Japanese family.

**Results:**

The proband exhibited advanced and metastatic gastric cancer, and was found to have a previously reported heterozygous frameshift variant in *CDH1* (NM_004360.3:c.1009_1010del:p.Ser337Phefs*12). Five at-risk relatives underwent presymptomatic molecular testing after careful genetic counseling, and three were molecularly diagnosed as positive for the variant. Esophagogastroduodenoscopy was performed in these relatives revealing abnormal small pale mucosal patches, small ulcerative lesion and no abnormal findings. Moreover, random and targeted biopsies were compatible with pathological diagnosis of HDGC in the three cases, all of which underwent total prophylactic gastrectomy.

**Conclusion:**

It is critical for the assessment and management of HDGC patients to be actively offered a multidisciplinary and familial-oriented approach. Notably, genetic screening in suspected individuals and familial members is a determining piece for a higher detection rate and the identification of clinical relevant mutations in both low and high-incidence gastric cancer countries**.**

## Introduction

Gastric cancer is the third highest cause of cancer death worldwide with over one million new cases diagnosed annually leading to more than 750 000 deaths [1]. Hereditary diffuse gastric cancer (HDGC) is a heritable form of gastric cancer [2–6]. Approximately 40% of families with HDGC were reported to have germline heterozygous pathogenic variants in *CDH1*, which encodes E-cadherin [2, 3, 7]. E-cadherin is a calcium-dependent cell membrane protein involved in cell–cell adhesion that is a known tumor suppressor [7]. The cumulative risk by age 80 years of diffuse gastric cancer in patients with *CDH1*-related HDGC is approximately 70% for men and 56% for women [2]. HDGC accounts for 1–3% of all gastric cancers [3, 4], and the majority of cases of *CDH1*-related HDGC reported to date have been in Western countries [8]. Despite the high incidences of gastric cancers in East Asian countries compared with Western countries, HDGC harboring *CDH1* variants has rarely been reported. Although several Japanese HDGC families with *CDH1* variant have been reported to date [9–14], awareness of this clinical entity remains low.

In the current report we present the clinical follow-up of three cases with HDGC harboring a previously described germline pathogenic variant (NM_004360.5:c.1009_1010del:p.Ser337Phefs*12), from a Japanese family with history of gastric cancer. These relatives were subjected to presymptomatic molecular diagnosis, followed by esophagogastroduodenoscopy with random and target biopsy and surgical intervention.

## Material and methods

Genetic counseling and DNA testing was provided to proband of this family, followed by to other members in this family. After informed consent was obtained, DNA was extracted from the peripheral blood leukocytes. First, genetic investigation was performed using PCR-direct sequence analysis for all the exons of *CDH1.* Next, a multiplex ligation dependent probe amplification (MLPA) analysis was performed. The family members found to have the genetic variant, careful examination by esophagogastroduodenoscopy including random biopsies was performed. A prudent follow-up has been done, not only for patients received total gastrectomy but also for other family members at risk for HDGC without initial approval for genetic testing.

This study was approved by the Shinshu University School of Medicine Biological and Medical Research Ethics Committee (approval No. 648) and Ethics Committee of Hamamatsu University School of Medicine (approval No. 20–011), respectively. The procedures used in this study adhere to the tenets of the Declaration of Helsinki. Written informed consent to be included in the study participate and publishing their images was obtained from all subjects.

### Case presentation

**Fig. 1:**
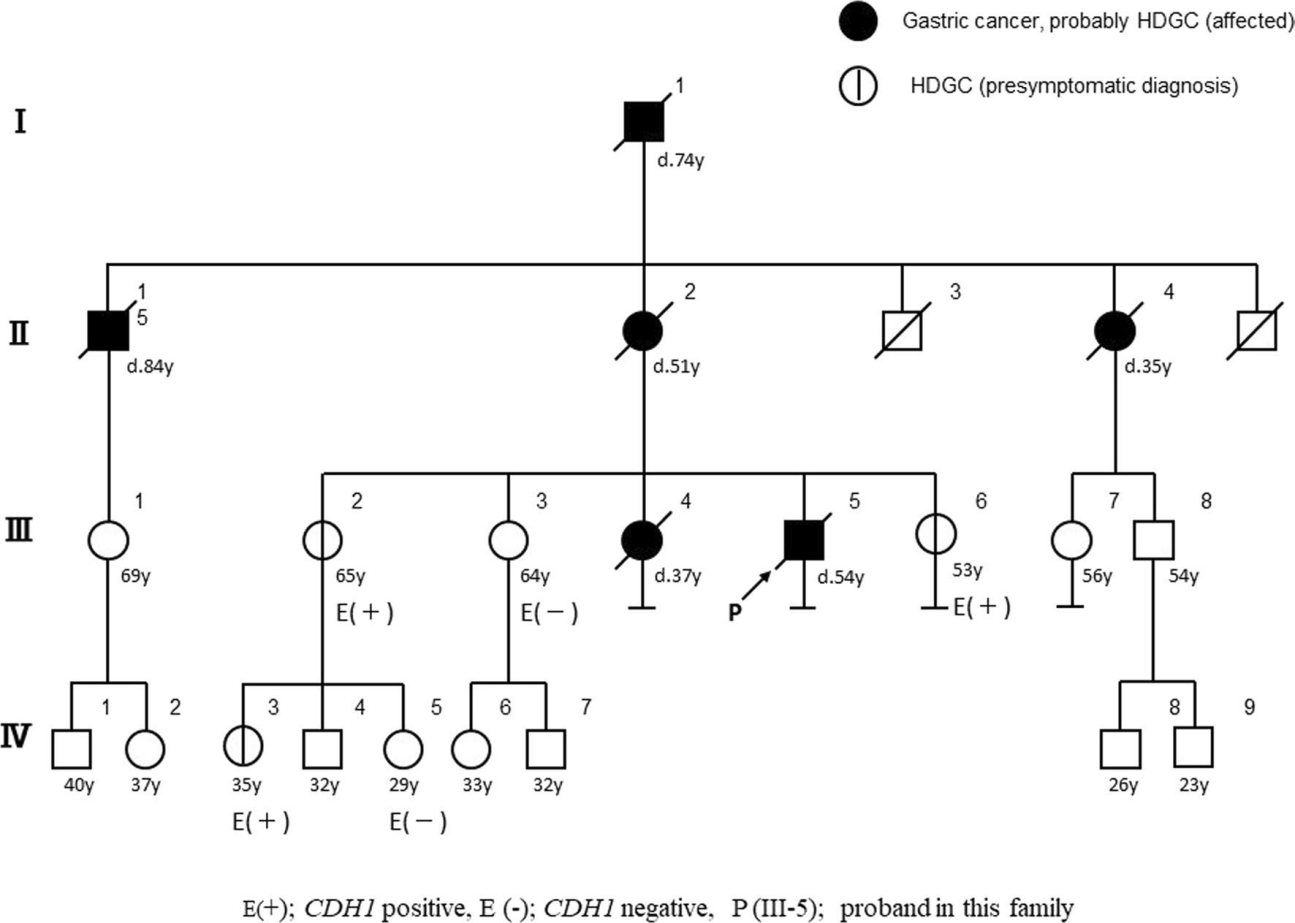
Pedigree of familial gastric cancer families with germline *CDH1* alterations. Discard E = *CDH1* genetic investigation, the meaning of E ( +); *CDH1* positive and E (−); *CDH1* negative. P indicated the proband (III-5) in this family

The pedigree of this family is shown in Fig. 1. The proband (III-5) was a 51-year-old man admitted to our hospital for further therapy due to postoperative gastric cancer recurrence. This patient underwent total gastrectomy with D2 lymphadenectomy 1 year prior to its admission. Pathological examination revealed adenocarcinoma, with pathological stage T4aN3M0 according to the Union for International Cancer Control (UICC) TNM Classification of Malignant Tumours, 8th Edition [15]. Adjuvant chemotherapy with tegafur/gimeracil/oteracil adjuvant chemotherapy was initiated, however, bowel obstruction due to peritoneal dissemination was observed 10 months after the initial surgery. The patient received chemotherapy for 8 months, but died due to uncontrollable progression of the disease at 53-year-old. His maternal grandfather (I-1) and aunt (II-4), mother (II-2), and elder sister (III-4) had all developed gastric cancer resulting in death. According to the International Gastric Cancer Linkage Consortium guidelines for HDGC families [2], the reported cases of gastric cancer point us to a family that fits the HDGC syndrome. After obtaining appropriate informed consent from this patient, genetic investigation was performed. A previously reported heterozygous frameshift variant in *CDH1* (NM_004360.3:c.1009_1010del:p.Ser337Phefs*12) was detected in the proband. After careful genetic counseling of at-risk relatives (multisession-based decision making, anticipatory guidance, and psychosocial considerations), three sisters (III-2, 3, 6) and two nieces (IV-3, 5) of the patient underwent presymptomatic molecular investigation. Three of these family members (III-2, 6, and IV-3) were found to have the variant.

### Case III-2

**Fig. 2:**
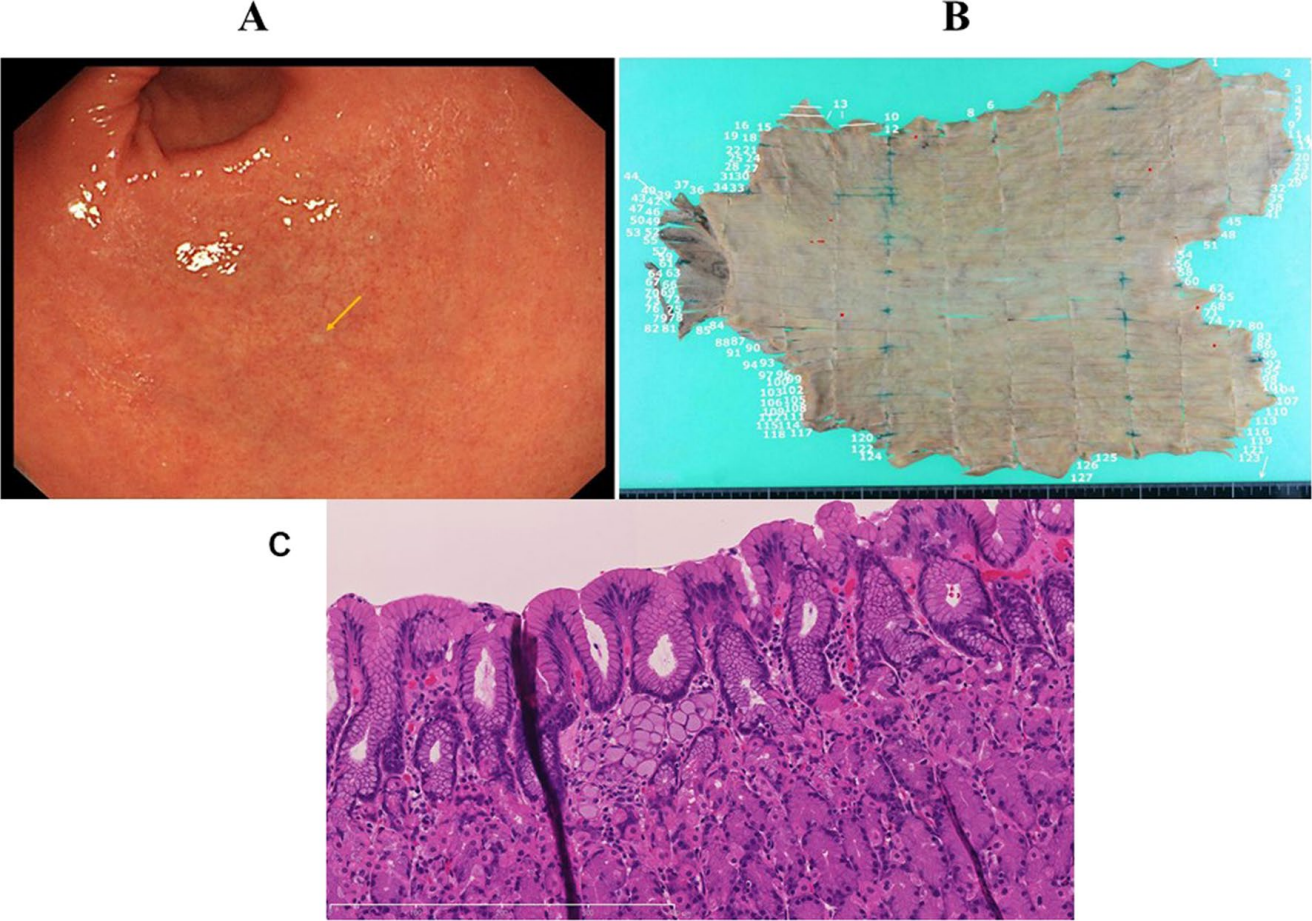
Esophagogastroduodenoscopy, post-operative specimen and pathological findings in case III-2. Esophagogastroduodenoscopy revealed a type0-IIb lesion in the gastric pyloric zone. There was white spot area (**A**, arrow). The gastrectomy mapping study revealed locations of early gastric cancer (**B**, red dots). The pathological examination showed intramucosal signet ring cell carcinoma (**C** × 20)

Case III-2 was 65-year-old female and had no prior history relevant to cancer. She underwent molecular diagnosis at age 62 years, and physical inspection showed no specific findings except operational scars in the abdomen associated with past surgery for appendicitis at age 14 years. Blood diagnosis revealed no abnormal finding, including tumor markers CEA (2.2 ng/mL) and CA19-9 (1.9 U/mL). Upper gastrointestinal endoscopy showed a small type 0-IIb lesion in the gastric pyloric zone (Fig. 2A). Histology of the biopsy specimens taken from the lesion indicated signet-ring cell carcinoma. The depth of cancer invasion was considered to be to the mucosal layer. Neither CT nor FDG-PET revealed any distant metastasis or lymph node involvement. Therefore, preoperative stage was considered to be T1a(M), N0, M0, and clinical stage was IA. She underwent laparoscopic total gastrectomy. There was no ascites or peritoneal dissemination. The gastrectomy mapping study revealed that a total of seven tumors with diameter < 1 mm were observed and pathological diagnosis of all the tumors was signet ring cell carcinoma (Fig. 2B and C). The depth of invasion was T1a(M) in all lesions. The number of dissected lymph nodes was 58 and no lymph node metastasis was found. According to the UICC TNM classification, a diagnosis of pT1aN0M0 stage IA was made. She had no recurrence at 47 months after the operation.

### Case III-6

**Fig. 3:**
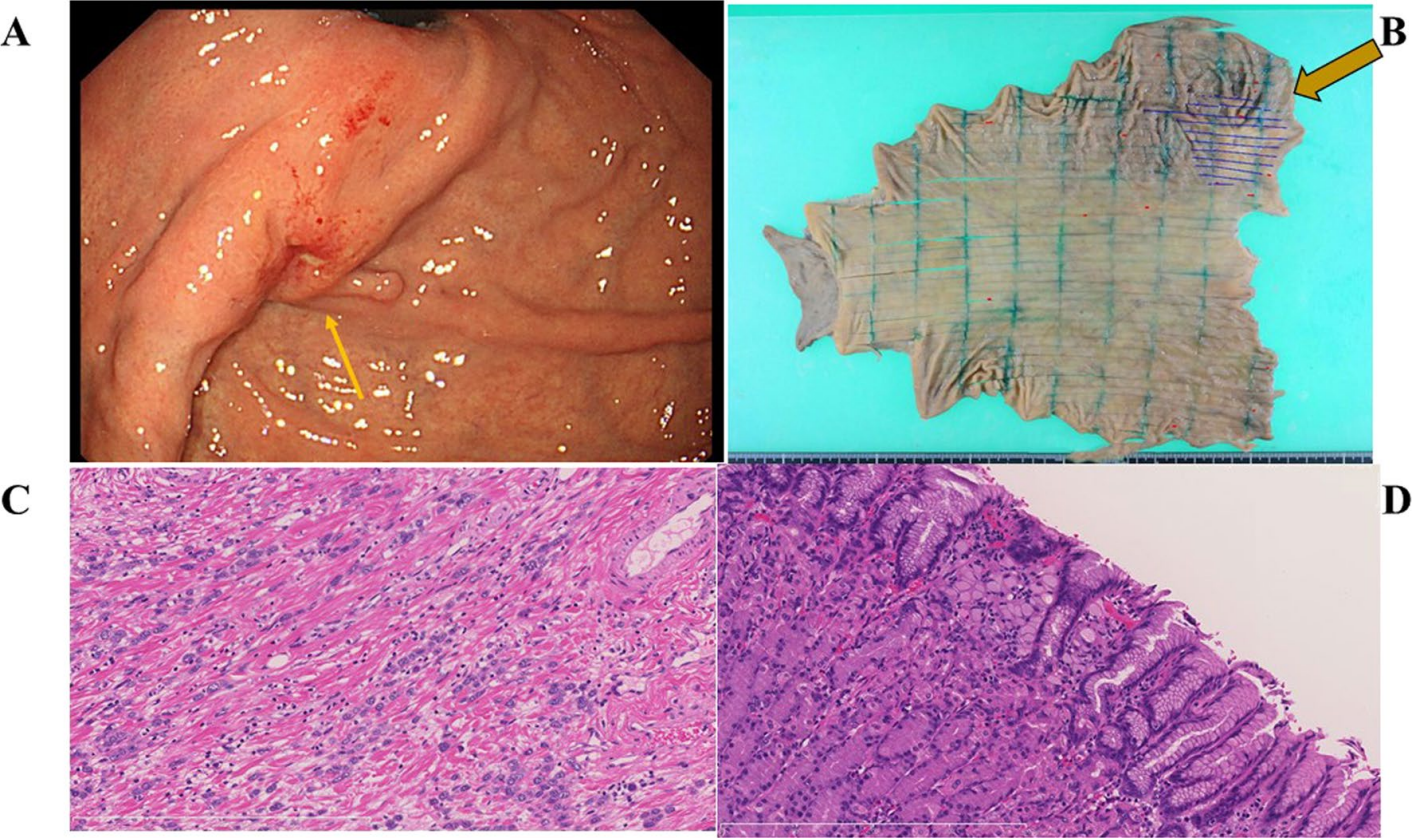
Esophagogastroduodenoscopy, post-operative specimen and pathological findings in case III-6. Esophagogastroduodenoscopy showed a type 0-IIa + IIc lesion at the gastric fundus with some ulceration in the center of the lesion (**A**). Resected specimen revealed a type 3 lesion with ulceration on the anterior wall of the gastric fundus (**B**, Arrow) and red dots indicated the presence of intramucosal signet-ring cells (**B**). Pathological findings revealed proliferating and infiltrating of poorly differentiated adenocarcinoma and signet ring cell carcinoma (**C**, × 20) and signet ring cell carcinoma under the mucosa (**D**, × 20)

Case III-6 was 53-year-old female and had no prior history relevant to cancer. She underwent molecular diagnosis at age 49 years, and there were no abnormal findings on physical or blood examination, including tumor markers carcinoembryonic antigen (CEA) 2.0 ng/mL (normal range, 0.0–3.4) and carbohydrateantigen 19–9 (CA19-9) 0.8 U/mL (normal range, 0.0–37.0). Esophagogastroduodenoscopy showed a type 0-IIa + IIc lesion at the gastric fundus with ulcers in the center of the lesion (Fig. 3A). Histology of biopsy specimens taken from the lesion indicated signet-ring cell carcinoma and poorly differentiated adenocarcinoma. The depth of cancer invasion was considered to be below the submucosal layer. Neither computed tomography (CT) nor ^18^F-fluorodeoxy glucose positron emission tomography (FDG-PET) revealed any distant metastasis or lymph node involvement. Therefore, preoperative stage was considered to be T1b (SM), N0, M0, with clinical stage IA. She underwent laparoscopic total gastrectomy. Laparoscopic findings revealed no ascites or peritoneal dissemination. The extent of lymph node dissection was D2. Macroscopically, the size of the tumor was 7.5 × 5.2 cm; the type 3 tumor was found in the upper back wall of the stomach (Fig. 3B, arrow); microscopically, the tumor was diagnosed as poorly differentiated adenocarcinoma (por2) and signet ring cell carcinoma (Fig. 3C and D), and the depth of tumor was T4a (serosal invasion); the number of dissected lymph nodes was 32, and one (# 2 lymph node) was found to have metastasis. According to the UICC TNM classification, the tumor was diagnosed as pT4N1M0 stage IIIA. In addition, a total of 20 lesions of intramucosal signet-ring cell carcinoma measuring about 2 mm were found in the stomach (Fig. 3D). Capecitabine plus oxaliplatin was given as adjuvant chemotherapy for 6 months, resulting in recurrence-free survival for 48 months.

### Case IV-3

**Fig. 4:**
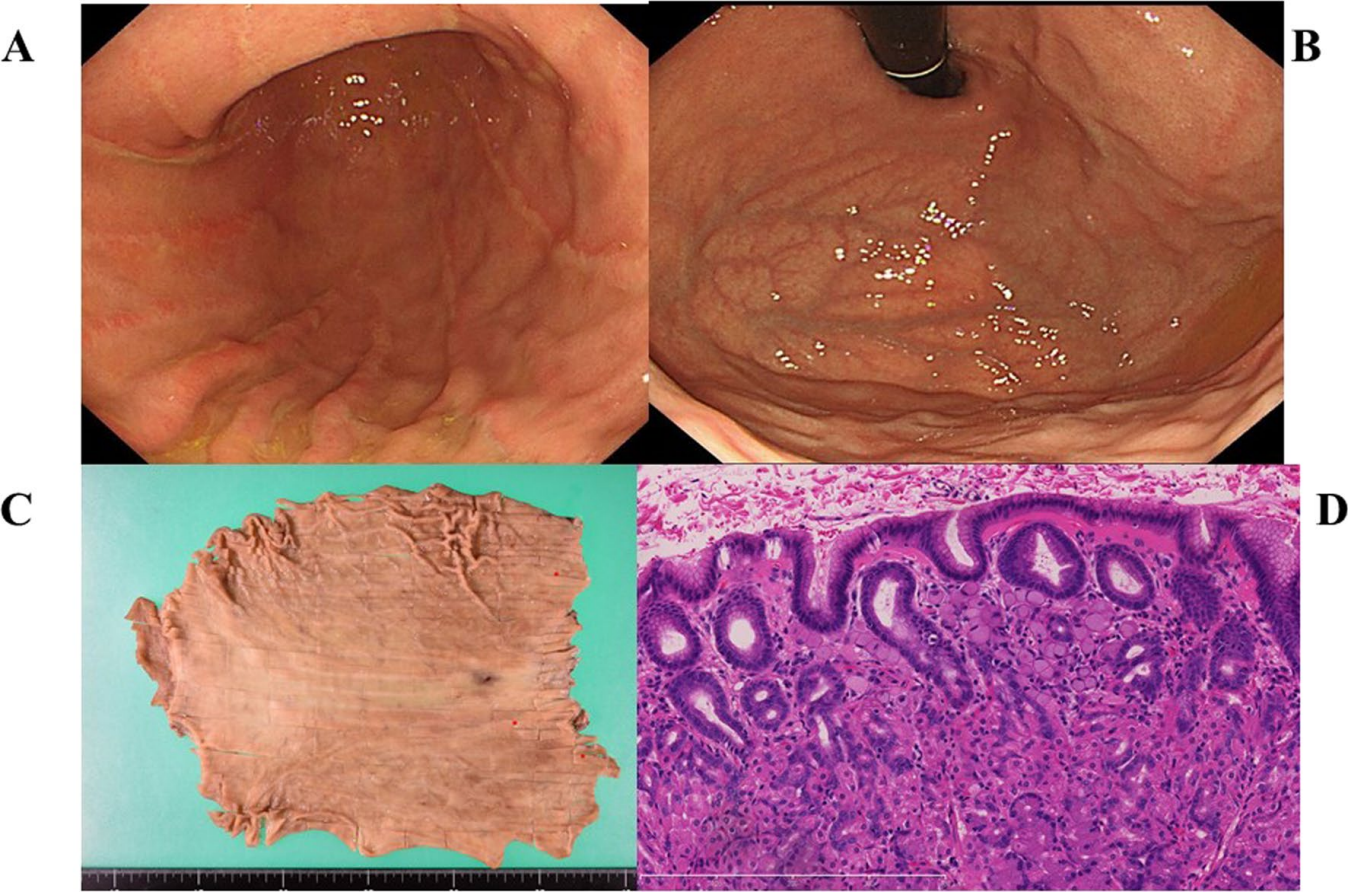
Esophagogastroduodenoscopy, post-operative specimen and pathological findings in case IV-3. Esophagogastroduodenoscopy showed no abnormal findings on gastric mucosa (**A** and **B**). The gastrectomy mapping revealed the locations of early gastric cancer (**C**, red dots). The pathological finding showed intramucosal signet ring cell carcinoma (**D**, × 20)

Case IV-3 was a 36-year-old female with no previous history relevant to cancer. She underwent molecular diagnosis at age 35 years, and physical and blood examination revealed no abnormal findings, including tumor markers CEA (2.7 ng/mL) and CA19-9 (1.4 U/mL). Esophagogastroduodenoscopy showed only superficial gastritis. Random biopsies were taken from 25 locations. Atypical cells were detected in one biopsy specimen, which was diagnosed as signet ring cell carcinoma. The depth of cancer invasion was considered to be the mucosal layer. Neither CT nor FDG-PET revealed any distant metastasis or lymph node involvement. Therefore, preoperative stage was considered to be T1a(M), N0, M0, and clinical stage was IA. She underwent laparoscopic total gastrectomy (Fig. 4A). There was no ascites and no peritoneal dissemination. A total of three tumors less than 1 mm in diameter were detected, and pathological diagnosis of all the tumors was signet ring cell carcinoma (Fig. 4B). The depth of invasion was T1a (M) in all lesions. The number of dissected lymph nodes was 39, and no lymph node metastasis was found. According to the UICC TNM classification, the diagnosis was pT1aN0M0 stage IA. She had no recurrence at 33 months after the operation.

## Discussion

In this study we reported a family with a medical history that meets the International Gastric Cancer Linkage Consortium criteria for HDGC families [2]. The genetic analysis revealed a frameshift variant (NM_004360.3:c.1009_1010del:p.Ser337Phefs*12) in *CDH1* gene that results in truncation of the E-cadherin protein. This variant was previously described by Benusiglio et al. [16] and registered as pathogenic in the available archive ClinVar (https://www.ncbi.nlm.nih.gov/clinvar/). Of note, although several Japanese families with *CDH1*-related HDGC have been reported [9–14], to our knowledge, this variant type was not identified and this is the largest number of family members with HDGC reported to date in Japan.

It is quite well known that serial screening gastroscopy is important for individuals with evidence of *CDH1* mutation [17–20]. Indeed, several studies showed that careful examination with targeted and random biopsies combined with detailed histopathology can identify early lesions allowing informed decision-making for gastrectomy [17–20]. Within the present family, histopathological diagnoses of HDGC were made in three at-risk family members, which led them to make an immediate decision for prophylactic surgery. Recent study from expert centers on HDGC surveillance endoscopy indicated increased detection rates of signet ring cancer cells in *CDH1* carriers [19, 20]. Thus, high-definition endoscopes, random biopsies and the experience of the endoscopist are important for screening gastroscopy.

Consequently, relatively good survival was obtained in all three cases without recurrence. However, we think that a mismatch between pre- and postoperative stage in case III-6 could imply the manifestation in HDGC. While the preoperative diagnosis was T1b (sm), N0, M0 with clinical stage IA indicating early cancer, operative observations revealed an unexpectedly advanced tumor, accompanied by lymph node metastasis 7.5 cm × 5.2 cm in diameter with serosal involvement (T4a). Therefore, adjuvant chemotherapy (capecitabine plus oxaliplatin) was introduced in this case. Considering the advanced lesions in postoperative specimens in case III-6, surveillance alone would not be sufficient, and prophylactic total gastrectomy could be a rational intervention for better prognosis in HDGC. Taken together, we intend to make presymptomatic diagnosis and perform early surveillance and intervention in patients with suspicion of familial gastric cancer.

Based on the international guidelines for HDGC syndrome [2], prophylactic total gastrectomy is recommended as the only life-saving approach for carriers of *CDH1* pathogenic variants. It is advised that this surgical intervention be performed in early adulthood up to around 30 years of age [21]. However, the optimal timing of surgical intervention remains to be determined. In addition, in Japan, prophylactic gastrectomy without a diagnosis of cancer is problematic with regard to not only insurance, but is also an ethical issue. Prophylactic total gastrectomy in young patients requires preoperative understanding of the patient based on adequate informed consent and counseling, as well as appropriate postoperative follow-up. Muir et al. [21] performed a prospective cohort study to examine the decision-making process of subjects undergoing prophylactic total gastrectomy, as well as physical and psychosocial outcomes, and described persistent mild physical symptoms affecting long-term quality of life [22]. Therefore, we emphasized that a multidisciplinary team involving surgeons, dieticians, genetic counselors, and specialist nurses would be necessary. Furthermore, as HDGC families are extremely rare in Japan, in view of public opinion against prophylactic gastrectomy, it may be necessary for academic societies to take the initiative in establishing treatment guidelines for prophylactic surgery.

In the present family, the age of death due to gastric cancer ranged from 35 to 84 years old. In general, the onset of the disease occurs at a young age. In other families with *CDH1* mutation including Japanese, most cases reported were in their 20 s or 30 s [4–14]. The age distribution in the present family may have been related to other factors contributing to the development of gastric cancer. It is well known that the major risk factor of gastric cancer is infection with *Helicobacter pylori* (*H. pylori*) [23], but the infection with *H.pylori* was negative in all three cases presented here. Furthermore, the variant type of *CDH1* in this family might be related the delayed onset of gastric cancer. At least, the type in this family was not reported in other Japanese HDGC [9–14]. Thus, further clinical experience and genetic background are required in subjects positive for *CDH1* mutation.

In summary, although HDGC has been regarded as rare in East Asia, we emphasize that *CDH1* germline mutation testing should be considered in patients with a familial history of gastric cancer, and careful examination and random biopsies using esophagogastroduodenoscopy are necessary in patients with suspicion of HDGC.

## Data Availability

All data analyzed during this study are included in this published article.
